# Clinical features and patency rates of Remedy® biodegradable peripheral stents

**DOI:** 10.5830/CVJA-2016-002

**Published:** 2016

**Authors:** Selma Kenar Tiryakioglu, Osman Tiryakioglu, Oguz Karahan, Sinan Demirtas, Fatih Gokalp, Kamuran Erkoc, Hakan Özkan, Ahmet Ozyazicioglu

**Affiliations:** Department of Cardiology, Bursa State Hospital, Bursa, Turkey; Department of Cardiovascular Surgery, Bahçeşehir University Medical Faculty, Istanbul, Turkey; Department of Cardiovascular Surgery, Dicle University Medical Faculty, Diyarbakir, Turkey; Department of Cardiovascular Surgery, Dicle University Medical Faculty, Diyarbakir, Turkey; Department of Cardiovascular Surgery, Şişli Etfal Education and Research Hospital, Istanbul, Turkey; Department of Cardiovascular Surgery, Medicalpark Bursa Hospital, Bursa, Turkey; Department of Cardiology, Bahçeşehir University Medical Faculty, Istanbul, Turkey; Department of Cardiovascular Surgery, Bursa Yüksek Ihtisas Education and Research Hospital, Bursa, Turkey

**Keywords:** biodegradable stent, peripheral vascular disease, clinical outcomes, patency rates, restenosis

## Abstract

**Objective:**

The aim of this study was to investigate the mid-term results of Remedy® biodegradable stents, which have recently come into use for lower-extremity arterial occlusive disease.

**Methods:**

Sixty-five patients, who underwent surgical intervention in various cardiovascular surgery clinics throughout Turkey, were included in the study. The total number of stents used was 92. The mean age of the patients was 64.11 ± 24.13 years (20–82), and 16 (24.6%) were female. The mean number of stents per patient was 1.42, and 70.7% of the lesions were TASC type A. Patients were followed for a mean of 32 months. Sixty-five patients underwent a control examination using either digital subtraction angiography or colour Doppler ultrasonography. In-stent restenosis was defined as ≥ 50% stenosis in the stent area in asymptomatic patients. The procedure was repeated if the degree of stenosis was ≥ 70%.

**Results:**

During the follow-up period, restenosis (≥ 50% stenosis) was observed in seven patients (10.7%). The patency rate after secondary intervention was 100%, and there was no loss of limbs in any patient. Restenosis was observed in six patients with superficial femoral artery stents, and in one patient with a popliteal arterial stent.

**Conclusion:**

Our experience shows that Remedy® biodegradable peripheral stents were safe and effective in our cohort of patients, with acceptable patency rates.

## Objective

The clinical use of stents for the treatment of peripheral artery disease is favoured for both long-term primary and secondary patencies, as well as shorter hospitalisation periods.^1^ The major stent types include bare-metal, nitinol, drug-eluting (for example, Sirolimus), ePTFE + nitinol (Viabahn) and biodegradable stents.^2^

Biodegradable stents contain the biologically degradable PLLA (poly-L-lactic acid) polymer. A few years after their implantation, biodegradable stents dissolve into water and carbon dioxide, and are absorbed by the arterial tissue.^3^-^5^ The development of restenosis in the stented segment does not limit the use of other procedures, and it is suitable for re-ballooning.^6^ In addition, when compared to metal stents, PLLA stents are more suitable as a platform for drug-releasing stents.^7^,^8^

This study investigated the mid-term results of Remedy® biodegradable stents.

## Methods

Sixty-five symptomatic patients were included in this study during a mean 32-month period between January 2010 and December 2014. The mean age of patients was 64.11 years (20–82). Biodegradable peripheral stents (Remedy®, Kyoto Medical, Japan) were used.

The stent-application procedure was performed in symptomatic patients who had ≥ 70% stenosis of the native artery diameter. An informed consent form was obtained from all patients prior to the procedure. Additionally, ethical approval was obtained from the local ethics committee of the University (2014-160).

An exclusive stent-application team was used to place the peripheral stents into appropriate patients (according to diameter of vessel and length of lesion) in different cities. Only selected lesions were studied due to the limited diameter (5–8 mm) and length (36 mm) of the stents.

Patients excluded from the study were those with long segments of stenosis, upper-extremity arterial interventions, large-diameter lesions, and if more than one stent was required. TASC type C and D patients who had had previous surgery and had appropriate vessel diameters and lesion lengths were included in the study. All patients were included in the study prospectively. In addition, some of the cases were re-evaluated retrospectively (especially patients with stent re-stenosis).

The peripheral arterial lesions of patients were classified in accordance with the TASC classification scheme.^9^ All procedures were performed under spinal or local anaesthesia. The anaesthesia protocol was selected according to patient compliance.

During the procedure, anticoagulation was provided by the administration of 100–300 U/kg of heparin. Following the procedure, 150 mg of clopidogrel (Plavix®, Sanofi, Turkey) was used as the anti-aggregant treatment protocol for patients who did not have any contraindications, and the treatment was subsequently continued with a dose of 75 mg/day.

The patients were monitored post-operatively in the first week, and second and sixth months. After one year, there were no significant clinical symptoms in any of the 43 patients. The mean follow-up period was 32 months. Digital subtraction angiography (DSA) was used to examine nine randomly selected patients, whereas colour Doppler ultrasonography (USG) was used to examine 52 randomly selected patients. Four symptomatic patients were evaluated separately.

## Statistical analysis

For analysis of the demographic, pre-operative, operative and postoperative data, and comparison of the median ± t standard deviation results of the group, categorical data chi-squared tests were performed using the Statistical Package for the Social Sciences (SPSS Inc, Chicago, Illinois, USA) 17.0 statistics software; p < 0.05 was considered significant.

## Results

Twenty-seven patients had diabetes, 38 had hyperlipidaemia, and seven had chronic obstructive pulmonary disease. Fifteen patients underwent coronary bypass surgery or were diagnosed with coronary artery disease (Table 1).

**Table 1:** Laboratory data and vascular pathology of patients. Mean age ± SD = 64.11 ± 24.13 years

*Parameters*	*n*	*%*
Gender (M/F)	49/16	75.4/24.6
TASC type A lesion	46	70.7
TASC type B lesion	12	20.0
TASC type C lesion	5	8.3
TASC type D lesion	2	3.3
Diabetes mellitus	27	41.5
Hyperlipidaemia (LDL > 130 mg/dl or 3.37 mmol/l)	38	58.4
COPD	7	10.7
Coronary artery disease (diagnosed)	15	23

TASC: TransAtlantic Inter-Society Consensus; LDL: low-density lipoprotein cholesterol; COPD: chronic obstructive pulmonary disease.

Sixty-five patients were examined with DSA or colour Doppler USG. DSA was performed in all symptomatic patients, and Doppler USG was performed in all patients.

In-stent restenosis was defined as ≥ 50% stenosis in the stent area in asymptomatic patients. The procedure was repeated if the degree of stenosis was ≥ 70%. None of the nine asymptomatic patients who were examined with DSA had ≥ 50% lesions. Three patients who were examined with colour Doppler USG had 50–70% lesions (two patients had a superficial femoral artery lesion; one had a popliteal arterial stent). None of the patients had ≥ 70% lesions.

In total, 46 patients had type A lesions, 12 had type B lesions, five had type C lesions, and two had type D lesions, according to the TASC classification scheme.^9^ Sixteen out of 65 patients (24.6%) were female and 49 (75.4%) were male (Table 1).

The total number of stents used was 92, and the mean number of stents per patient was 1.42.^1^-^3^ The mean stent length was 53 mm per patient (36–108 mm). Twenty-two stents (24%) were implanted into the right superficial femoral artery, 31 (33.7%) into the left superficial femoral artery, 14 (15.2%) into the left iliac artery, 17 (18.4%) into the right iliac artery, five (7.8%) into the left popliteal artery, and three (3.2%) into the right popliteal artery. The size of the stents ranged between 5 and 8 mm; the most commonly used stent width was 6 mm.

The procedure was performed under spinal anaesthesia in five patients (7.7%) and local anaesthesia in 60 patients (92.3%) (Table 2.). There was 100% success rate of the procedure. In all patients, it was possible to palpate the pulse with a hand on the distal part of the stent zone after the procedure. There was no limb loss in any patient.

**Table 2:** Stent features and sites for stent therapy in patients

*Stents*	**
Total number of stents (n)	92
Mean stent diameter (mean ± SD mm)	6.9 ± 1.3
Stent length per patient (mean ± SD mm)	53.0 ± 14.6
Restenosis (n/%)	7/7.6
Application area (n/%)	
Superficial femoral artery	53/57.6
Iliac artery	31/33.7
Popliteal artery	8/8.7
Anaesthesia (n/%)	
Local	60/92.3
Spinal	5/7.7

The mean follow-up period was 32 months (18–48 months), and in-stent restenosis developed in three patients. These patients received a secondary balloon dilatation procedure. The superficial femoral artery stent was fully occluded in one patient, and the patient underwent femoropoliteal bypass. The remaining patients have had no complications from the stenting.

During the follow-up period, DSA was performed in all patients who were admitted symptomatically (four patients). Total occlusion was observed in one patient and 70–90% stenosis was observed in three patients (all patients had a superficial femoral artery stent) (Table 3). All patients were alive, so the Kaplan–Meier life curve was not included in the text, and we used single-group analysis.

**Table 3:** Clinical imaging and severity of stenosis according to symptoms

	*50% stenosis*	*50–70% stenosis*	*≥ 70% stenosis*
Symptomatic patients: DSA (n = 4)	-	-	4*
Asymptomatic patients: DSA (n = 9)	9	-	-
Asymptomatic patients: USG (n = 52)	49	3**	-
Total	58	3	4

*All were superficial femoral artery (SFA) lesions.

**Two patients had SFA stents; one had a popliteal artery stent.

USG: ultrasonography; DSA: digital subtraction angiography.

## Discussion

This was a unique study demonstrating the results of mid-term peripheral application of biodegradable stents. The findings of this study show that biodegradable stents had successful clinical outcomes during an average of 32 months’ follow-up period. It appears from this study that biodegradable stents have good mid-term patency rates in peripheral arterial occlusions.

In patients with limb ischaemia, the first-line approach for revascularisation has shifted over the past decade from bypass surgery to endovascular intervention.^10^ Stenting for the treatment of lower-extremity arterial occlusive disease is an important tool and continues to evolve, with new stent designs and technologies being developed to provide superior patency rates and limb salvage.^11^,^12^

Short- and long-term outcomes of peripheral artery stents are available for metal, nitinol and e-PTFE-coated nitinol stents. Initially, efforts were directed at overcoming the negative outcomes of metal stents, especially in long lesions.^13^ Owing to the recent advances in stent technology, biodegradable stents have been produced to avoid the undesirable outcomes of classic stents, which lead to mechanical restenosis with stent fracture.

This technology has been extensively evaluated in the coronary artery system, but not in peripheral arteries. The outcome of bio-absorbable stents remains unclear.^14^ We therefore investigated the patency rates of this technology in the peripheral arterial system.

Outcomes of even classical stents have not been sufficiently documented. Short hospitalisation times, successful outcomes in stent patency, higher secondary patency rates, and comparable outcomes with grafts have increased the popularity of peripheral stent applications in TASC type A and B lesions. The primary and secondary patency rates of PTFE-coated stents in veins above the knee have equal efficacy to PTFE grafts, and are comparable with that of saphenous veins. Formation of the neo-intima layer in closed stents prevents the development of restenosis in the early period, and ensures that stents remain patent for a longer period.^15^,^16^

The use of stents for suitable lesions, especially in the case of iliac artery lesions, protects the patient from possible abdominal surgery and increases the incidence of long-term patency as the vein diameter is also suitable. In the present study, stenosis was not observed during the follow-up period after iliac artery stent implantation.

The PLLA peripheral stent is the first fully bioresorbable stent to be implanted in humans, with complete degradation taking 12 to 24 months. This resulted in less vessel wall injury during implantation and therefore less initial thrombus formation and reduced intimal hyperplasia.

The existing literature on the use of biodegradable peripheral stents on coronary arteries is limited, and their superiority over drug-releasing stents has not been proven to date. The existing data regarding the use of biodegradable peripheral stents in peripheral arteries is insufficient.^17^

The application of biodegradable peripheral stents has positive contributions to the collateral system in particular. The collateral system in the application area does not close, but rather increases in the long term, and supports development of the existing systems.^18^ Collateral closure was not observed during the procedure in our patient group, and we also observed that these veins stayed open in the patient group examined with DSA.

The major advantages of using biodegradable stents include practical application, a suitable structure for secondary interventions, their absorbable and non-depositing nature, perfect tissue compatibility, and a lower rate of restenosis in experimental studies. A feature of the biodegradable stent is that it allows for re-intervention due to its structure (after implantation of the stent, integration occurs between the stent structure and vessel wall). Therefore re-intervention can easily be carried out (balloon and stent or surgery) in TASC type A lesions when they become TASC type C or D. Another advantage is that there is no possibility of stent fracture, which eliminates anti-aggregant use. They are also suitable for use in patients with a metal allergy.

There are certain disadvantages, including insufficient number of clinical studies investigating long-term outcomes. Moreover, the length and diameter of the available stents are not suited for every clinical scenario.

The absence of a control group was the main limitation of this study. The ethics committee approved the study protocol, which was created with the use of a single type of stent to avoid bias and to provide similar opportunities for all patients. This pilot study was therefore conducted without a control group.

## Conclusion

In this study, primary implantation of a Remedy® biodegradable stent for moderate-length lesions in lower-extremity arterial occlusive segments of patients with claudication was associated with better mid-term results. Our experience shows that the Remedy® peripheral stents were safe and effective in our cohort of patients, with acceptable patency rates.
